# Circular RNA expression in isoproterenol hydrochloride-induced cardiac hypertrophy

**DOI:** 10.18632/aging.102761

**Published:** 2020-02-05

**Authors:** Ming-Hui Yang, Hao Wang, Sheng-Na Han, Xin Jia, Si Zhang, Fei-Fei Dai, Meng-Jiao Zhou, Zhongnan Yin, Tian-Qi Wang, Ming-Xi Zang, Li-Xiang Xue

**Affiliations:** 1 Department of Biochemistry and Molecular Biology, School of Basic Medical Sciences, Zhengzhou University, Zhengzhou City, Henan 450001, P. R. China; 2 Medical Research Center, Peking University Third Hospital, Beijing 100191, P. R. China; 3 Department of Pharmacology, School of Basic Medical Sciences, Zhengzhou University, Zhengzhou City, Henan 450001, P. R. China; 4 School of Pharmaceutical Sciences, Zhengzhou University, Zhengzhou City, Henan 450001, P. R. China; 5 Biobank of Peking University Third Hospital, Beijing 100191, P. R. China

**Keywords:** cardiac hypertrophy, circRNAs, isoproterenol hydrochloridex

## Abstract

Circular RNA (circRNA) is a novel class of noncoding RNAs, and the roles of circRNAs in the development of cardiac hypertrophy remain to be explored. Here, we investigate the potential roles of circRNAs in cardiac hypertrophy. By circRNA sequencing in left ventricular specimens collected from 8-week-old mice with isoproterenol hydrochloride-induced cardiac hypertrophy, we found 401 out of 3323 total circRNAs were dysregulated in the hypertrophic hearts compared with the controls. Of these, 303 circRNAs were upregulated and 98 were downregulated. Moreover, the GO and KEGG analyses revealed that the majority of parental gene of differentially expressed circRNAs were not only related to biological process such as metabolic process and response to stimulus, but also related to pathway such as circulatory system and cardiovascular diseases. On the other hand, total 1974 miRNAs were predicted to binding to these differentially expressed circRNAs, and the possible target mRNAs of those miRNAs were also predicted and analyzed in terms of functional annotation. Finally, we identified that ANF and miR-23a are downstream targets of circRNA wwp1, suggesting that circRNA wwp1 exerts inhibitory roles of cardiac hypertrophy via down-regulation of ANF and miR-23a, which underlying the potential mechanisms whereby circRNA regulates cardiac hypertrophy.

## INTRODUCTION

Cardiac hypertrophy is an adaptive response to various physiological and pathologic stimuli; however, chronic, prolonged stress will ultimately lead to irreversible pathological hypertrophy, accompanied by myocardial dysxfunction, and eventually heart failure [1]. Accordingly, cardiac hypertrophy is a well-recognized risk factor for cardiovascular disease, but no molecular targets for convincingly preventing or treating cardiac hypertrophy have yet been found, most likely because our understanding of the molecular mechanisms underlying cardiac hypertrophy remains incomplete [2].

In recent years, intracellular signaling pathways regulating cardiac hypertrophy have been explored, including ERK1/2, AMPK, and protein kinase C [3]. Among the signaling pathways, phosphatidylinositol-3 kinase (PI3K)-Akt is the main pathway because it account for an increase in protein synthesis during cardiac hypertrophy [4]. Alternatively, metabolic alterations are also investigated because there is an energy substrate shift from fatty acid to glucose accompanied pathological cardiac hypertrophy [4]. Unfortunately, many attempts have been made on signaling pathways and metabolic alterations, but the function of noncoding RNAs in cardiac hypertrophy deserves more attention.

Indeed, recent studies have revealed that small noncoding RNAs, which constitute a major epigenetic control mechanism, may be able to mediate the development of cardiac hypertrophy by targeting signaling molecules involved in cardiac hypertrophy, as well as fetal gene reprogramming [1, 5]. Circular RNAs (circRNAs) represent a class of small noncoding RNAs that control gene expression at various levels, including at transcription initiation and posttranscription [6]. In the heart, circRNAs might be involved in the development of cardiac hypertrophy by functioning as microRNA molecular sponges [5, 7]. Although emerging evidence suggests that circRNAs play essential roles in the initiation and development of cardiac hypertrophy, the understanding of the circRNA landscape in the hypertrophic heart and its effects on cardiac hypertrophy subjected to various stimuli is still quite limited [5, 8, 9].

To explore the expression profile and potential functions of circRNAs in cardiac hypertrophy, the present study was designed to determine whether alterations in circRNA expression occur in cardiac hypertrophy induced by isoproterenol hydrochloride. Here, we show that isoproterenol hydrochloride significantly affects circRNA expression in the hypertrophic heart of mice. Furthermore, miRNAs binding to these differentially expressed circRNAs, and its possible target mRNAs were predicted and analyzed in terms of functional annotation. Our study indicated that the circRNAs might have possible functional implications in cardiac hypertrophy, which set the stage for the use of circRNAs as new therapeutic targets for cardiac hypertrophy.

## RESULTS

### Identification of circRNA expression profiles

**Figure 1:**
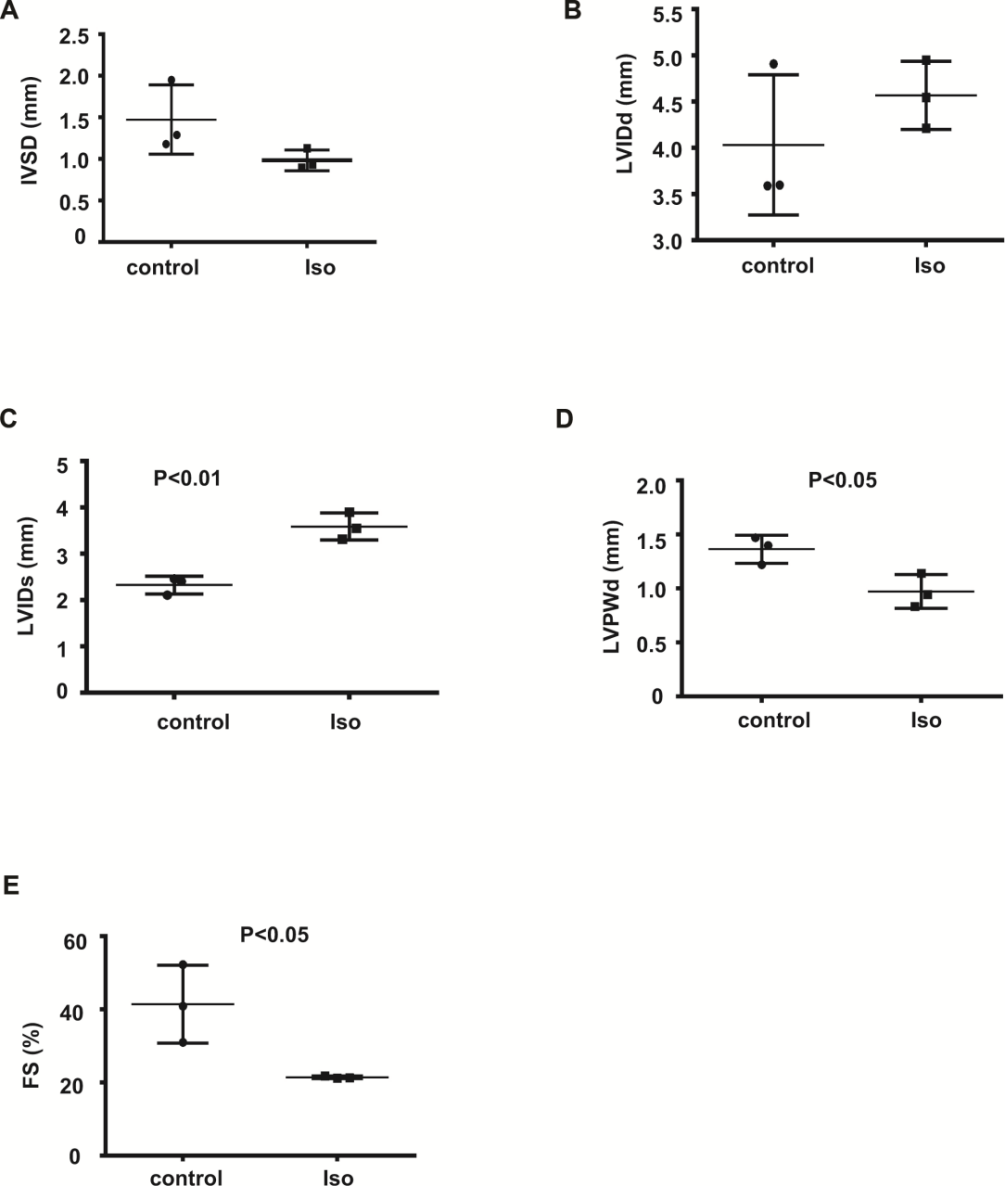
**Isoproterenol treatment resulted in decreased cardiac function. Cardiac function was determined by echocardiography in 8-week-old mice infused with isoproterenol.** The interventricular septum thickness at end-diastole (IVSD) (**A**) end-diastolic and end-systolic left ventricular internal diameter (LVIDd and LVIDs) (**B**, **C**) end-diastolic left ventricular posterior wall thickness (LVPWd) (**D**) and fractional shortening (FS) (**E**) were determined from the M-mode images. ctr: control, Iso: isoproterenol. Data are the mean ± SEM. n= 3 per group.

To determine the expression profiles of circRNAs during cardiac hypertrophy, 8-week-old mice were infused with isoproterenol for 14 days to induce cardiac hypertrophy; then, echocardiography was performed to evaluate the functional alterations following the isoproterenol-induced cardiac hypertrophy response. The isoproterenol-treated mice showed a decrease in the IVSD, LVPWd, and FS and an increase in the LVIDd and LVIDs relative to the controls (Figure 1A–1E), suggesting that isoproterenol efficiently induced cardiac hypertrophy.

**Figure 2:**
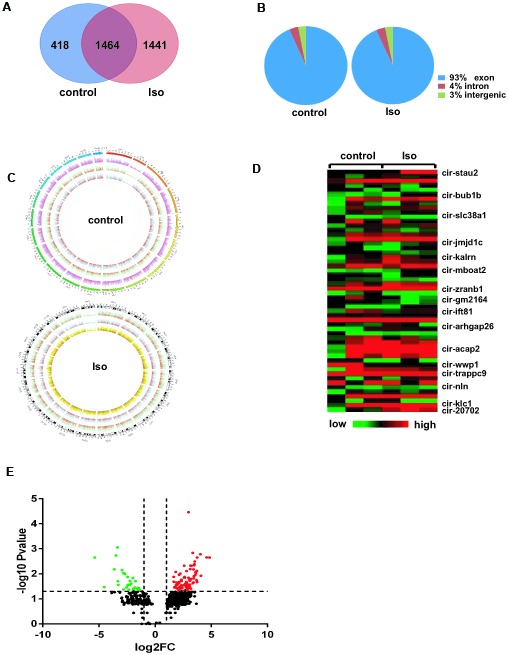
**Differentially expressed circRNAs in left ventricles after isoproterenol treatment.** (**A**) Venn diagrams showing the overlap of 1464 circRNAs between the two experimental groups. (**B**) Genomic origin of circRNAs obtained from alignment to the mouse genome. (**C**) Circos visualization of the distribution of the identified circRNAs in mouse chromosomes. The outermost layer represents all mouse chromosomes, and each of the 3 inner layer show the circRNA distribution of every sample in the same group, while the red represents the expression levels of the circRNAs. (**D**) Heatmap of circRNA expression determined in the left ventricles of mice with or without isoproterenol treatment, as determined by next-generation sequencing (NGS). (**E**) Volcano plot showing altered circRNA expression upon isoproterenol treatment. A 2-fold change and P<0.05 were considered significant, and significantly upregulated and downregulated circRNAs are represented by red and green dots, respectively.

Next, after 14 days of treatment with isoproterenol, circRNAs were characterized in left ventricular specimens collected from the control and isoproterenol-treated mice. Venn diagrams showed that 1441 circRNAs were specific to ventricles treated with isoproterenol, while 1464 circRNAs were expressed in both experimental groups (Figure 2A). Interestingly, the percentage of circRNA sequences that could be matched to the exon, intron, and intergenic regions were the same between the control and isoproterenol-treated ventricles, at 93%, 4%, and 3%, respectively (Figure 2B). Besides, these circRNAs are widely distributed in all chromosomes but not the mitochondrial chromosomes, and the number of circRNAs in different chromosomes and the densities of circRNAs in different chromosomal regions were also different (Figure 2C).

### Altered circRNA profiles induced by isoproterenol

Based on the circRNA expression results, we next analyzed the differential expression of circRNAs between ventricles without or with isoproterenol treatment. Among the 1464 circRNAs commonly expressed in ventricles with and without isoproterenol treatment, 119 circRNAs were significantly (P<0.05) differentially expressed compared with the untreated ventricles; 89 circRNAs were upregulated, and 30 were downregulated. In contrast to the commonly expressed circRNAs, 1441 circRNAs were expressed only in ventricles with isoproterenol treatment but not without isoproterenol treatment, of these, 214 circRNAs were significantly (P<0.05) upregulated, and the most upregulated expression level was 228.7 spliced reads per billion mapping (SRPBM) (Supplementary Table 1). Similarly, for the 418 circRNAs expressed only in untreated ventricles, 68 circRNAs were significantly (P<0.05) downregulated, and the most downregulated expression level was from 24.1 to 0 SRPBM (Supplementary Table 1), some of these altered circRNAs were presented in a heatmap and volcano plot (Figure 2D–2E).

### Q-PCR validation of differentially expressed circRNAs

**Figure 3:**
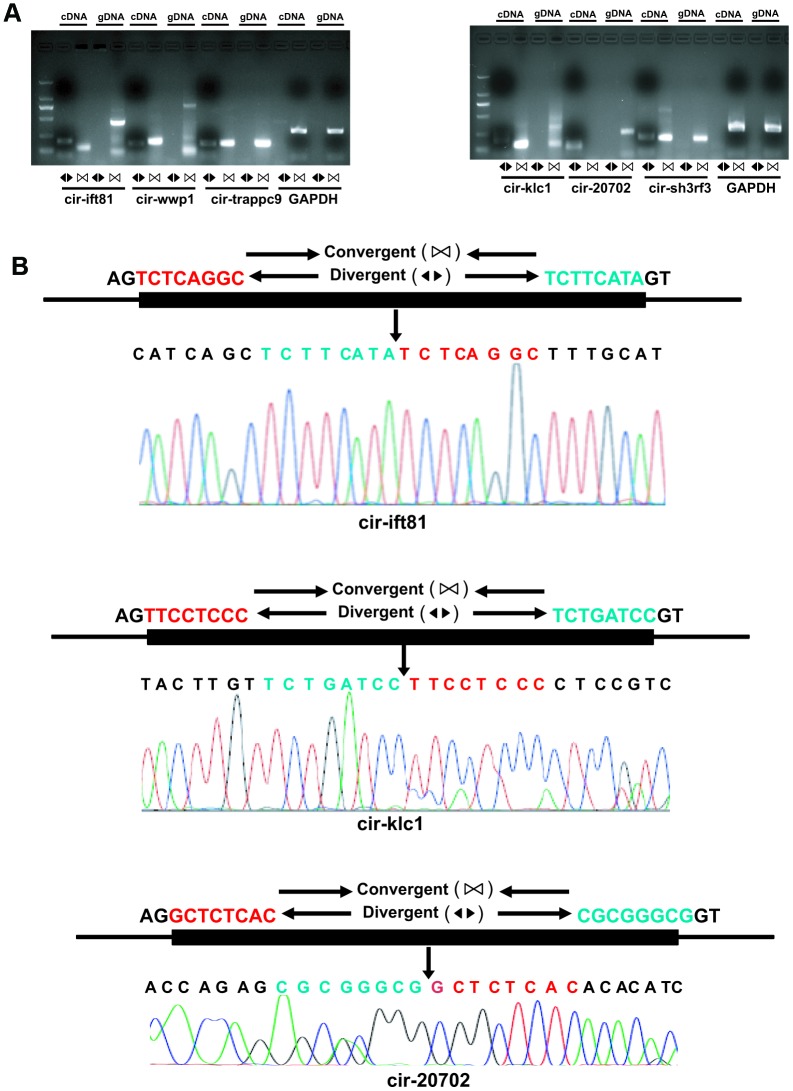
**Validation of altered circRNA expression.** (**A**) RNA was extracted from the ventricles of mice treated with or without isoproterenol (Iso) and the circular RNAs were validated by Real-time PCR and Sanger sequencing. The divergent primers were used to amplify circRNAs but the convergent primers were designed to detect the linear form both in cDNA and genomic DNA (gDNA), GAPDH is as a linear control. (**B**) The amplified circRNAs were confirmed to be head-to-tail spliced via sequencing.

**Figure 4:**
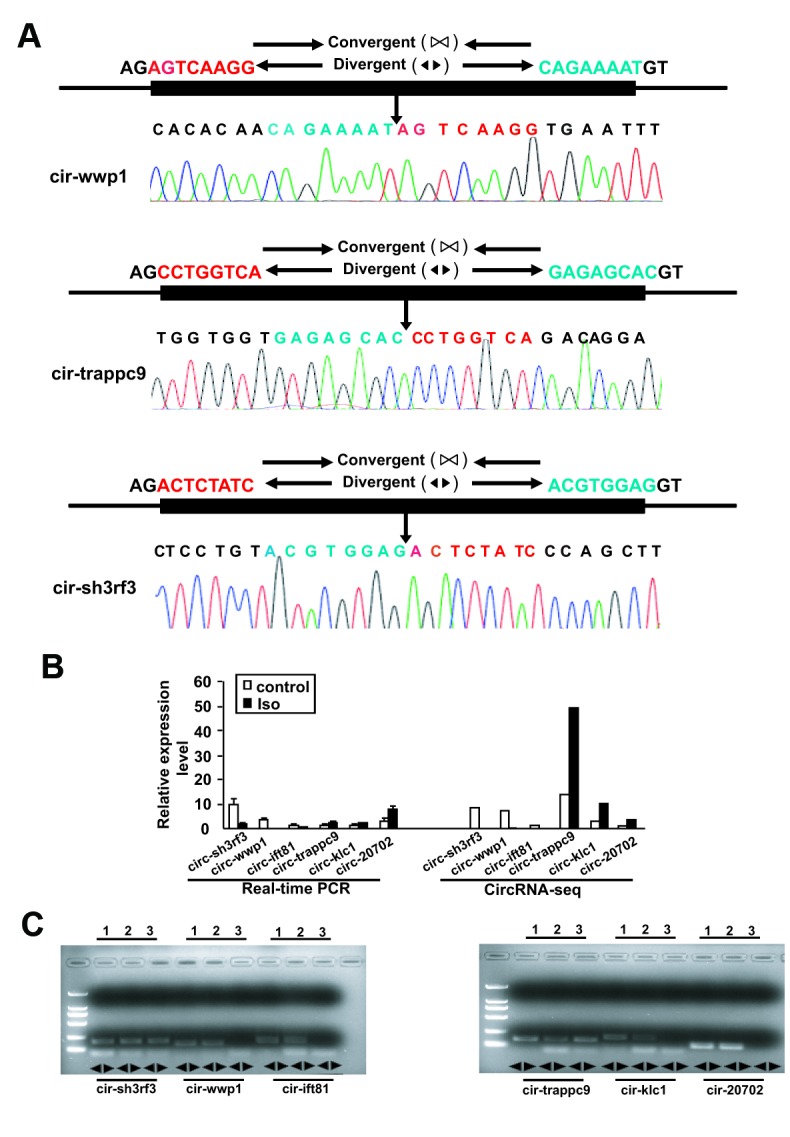
**Validation of altered circRNA expression by qRT-PCR.** (**A**) The amplified circRNAs were confirmed to be head-to-tail spliced via sequencing. (**B**) The expression levels of differentially expressed circRNAs were determined by Real-time PCR. The Data are means ± SEM with 3 mice per group. Seq: sequence. (**C**) The expression of circular RNAs in human skeletal muscle cells and the liver of mice. Lane 1, 2, and 3 represented the RNA in heart, liver and human skeletal muscle cells, respectively.

To validate the reliability of altered circRNAs obtained by next-generation sequencing, several circRNAs, including 6 circRNAs (mmu_circRNA_sh3rf3, mmu_ circRNA_wwp1, mmu_circRNA_ift81, mmu_circRNA_ trappc9, mmu_circRNA_klc1, and mmu_circRNA_ 20702) were randomly selected for validation of their expression by Q-PCR. To ensure the specific amplifications of circular RNA, divergent primers were designed while a pair of convergent primers were used as a control. The Q-PCR results showed that amplification products for all circRNAs from divergent primers were consistent with the expected products, and sanger sequencing further confirm the sequences (Figures 3 and 4A). Furthermore, the relative expressions of mmu- circ sh3rf3, mmu- circ wwp1 and mmu- circ ift81 were also significantly lower in ventricles treated with isoproterenol than those untreated ventricles (Figure 4B), whereas the expression levels of circRNAs 20702, trappc9 and klc1 in isoproterenol treatment ventricles were higher than those untreatment ventricles (Figure 4B), which further demonstrated the results obtained from next-generation sequencing. To address the circular RNAs conservation, we checked the circular RNA expression in human skeletal muscle cells and liver of mice, as shown in Figure 4C, we found that circ-klc1, circ-20702, circ-ift81, and circ-wwp1 are not expressed in human, suggesting that these circular RNAs are not conserved. Whereas circ-sh3rf3, and circ-trappc9 are expressed in human, suggesting that these circular RNAs are conserved in human. On the other hand, all the circular RNAs are expressed both in heart and liver of mice (Figure 4C), suggesting that these circular RNAs are not tissue-specific expression.

### Gene Ontology (GO) and KEGG pathway analyses of differentially expressed circRNAs

**Figure 5:**
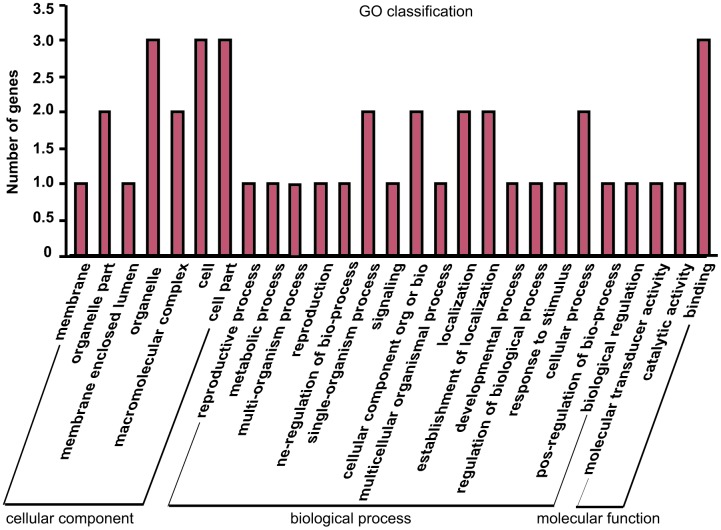
Gene Ontology (GO) annotation of parental genes of the differentially expressed circRNAs between isoproterenol- treated and untreated mice.

The parental gene of differentially expressed circRNAs may be related to the potential function of the circRNAs, so GO analysis were performed to annotate the function of the parental genes of differentially expressed circRNAs. The results showed that the genes are involved in cellular component, molecular function, and biological process (Figure 5). Meanwhile, the majority of genes were related to biological process, such as metabolic process, biological regulation, and response to stimulus, which indicated that the altered expressed circRNAs from isoproterenol treatment may play roles in these process to response the hypertrophic stimulation.

**Figure 6:**
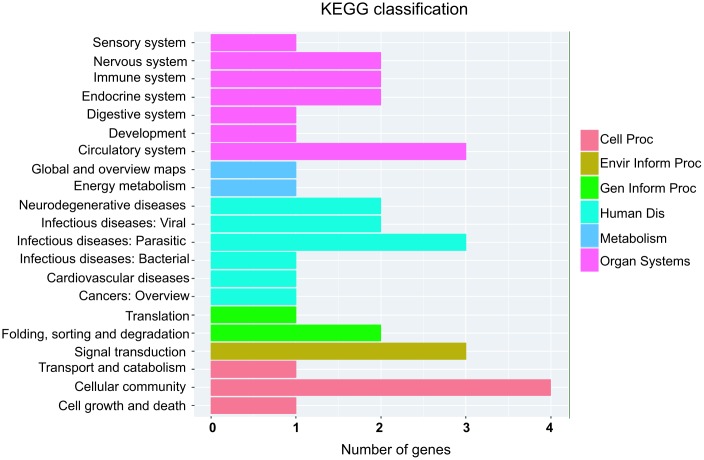
KEGG pathway annotations of the organismal systems (Organ Systems), human diseases (human Dis), cellular processes (cell Proc), environmental information processing (Envir Inform Proc), genetic information processing (Gen Inform Proc) and metabolism pathways. KEGG: Kyoto Encyclopedia of Genes and Genomes.

Next, KEGG pathway were employed to analyze the potential pathway of parental gene of differentially expressed circRNAs. Interestingly, circulatory system and cardiovascular diseases were involved into the altered expressed circRNAs (Figure 6). Moreover, energy metabolism and signal transduction pathway, which may be account for cardiac hypertrophic response induced by isoproterenol hydrochloride, are also related to the differentially expressed circRNAs (Figure 6), these results indicated that the differentially expressed circRNAs may exert their effects through these pathways.

### Delineation of binding miRNAs of altered expressed circRNAs

**Figure 7:**
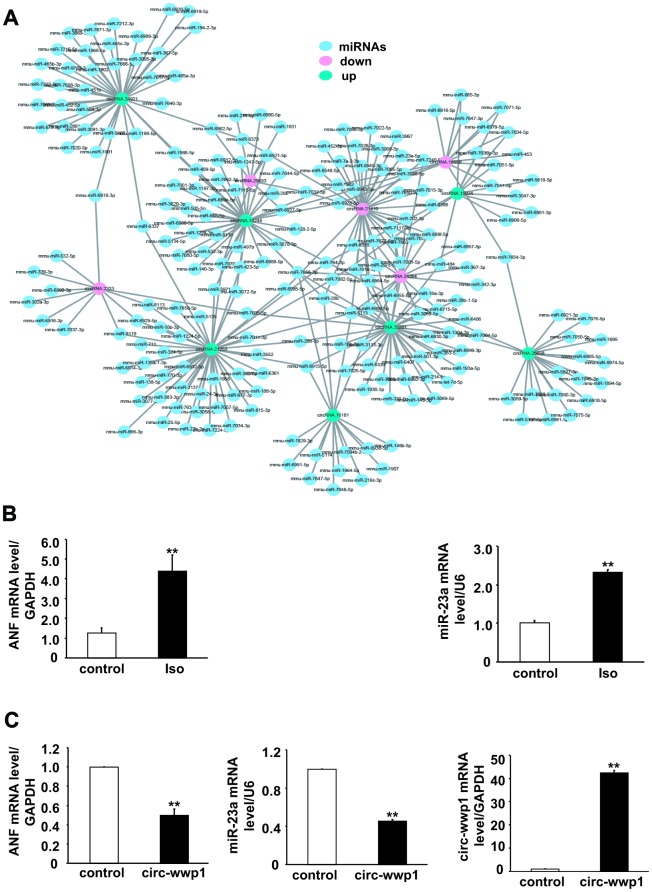
**CircRNA-miRNA interaction network for differentially expressed circRNAs in isoproterenol- treated and untreated mice.** (**A**) Red circles represent down-regulated circRNAs, green circles represent up-regulated circRNAs, and blue circles represent binding miRNAs. (**B**) mRNA level of ANF and miR-23a were determined in left ventricles after isoproterenol treatment. Iso: isoproterenol. (**C**) mRNA level of ANF and miR-23a were determined in cardiomyocytes infected with adenovirus circ-wwp1, the results are the mean±SEM of three independent experiments. ** indicates p < 0.01 compared with control.

A possible mechanism by which circRNAs regulate gene expression is that circRNAs act as miRNA sponges to sequester its inhibitory effect on target genes. To confirm the differentially expressed circRNAs have the same function as those circRNAs previously reported, we predicted potential miRNA binding sites on circRNAs using miRanda database tool. Total 1974 miRNAs were predicted to binding to these differentially expressed circRNAs, and 7 up-regulated and 5 down-regulated circRNAs were selected for delineation of their interaction network by cytoscape software (Figure 7). The results showed that one circRNA could serves as sponges for many miRNAs, for example, the number of miRNAs which bind to mmu-circRNA stau2 is up to 33. On the other hand, one miRNA could be targeted by more circRNAs, as shown in Figure 7, mmu-miR-1966-5p was targeted by mmu-circRNA stau2 and mmu-circRNA crip2, these results indicated that there is a complicated interaction network between the differentially expressed circRNAs and their target miRNAs.

To address the potential targets, we infected with adenovirus circRNA wwp1 in cardiomyocytes, and extract RNA for detection the mRNA level of ANF and miR-23a, which are upregulated in hypertrophied heart. As shown in Figure 7B and 7C, the mRNA level of ANF and miR-23a is decreased in circRNA wwp1 over-expressed cardiomyocytes, suggesting that ANF and miR-23a are downstream targets of circRNA wwp1, and circRNA wwp1 exerts inhibitory roles of cardiac hypertrophy via down-regulation of ANF and miR-23, which underlying the potential mechanisms whereby circRNA regulates cardiac hypertrophy.

### Gene Ontology (GO) analyses of predicated target mRNAs of miRNAs

**Figure 8:**
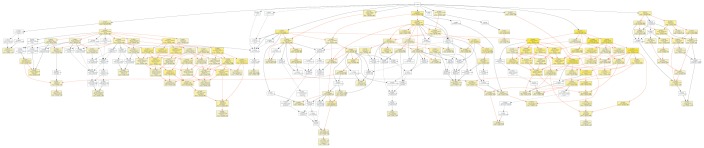
Diagrams of enriched GO terms of mRNAs targeted by miRNAs in the biological process category using GOEAST tools. yellow and white boxes indicated significantly and non-significantly enriched GO terms, respectively. More intense yellow colors means the more significant biological process terms, and different colors arrows represent relationships between two enriched (red) or unenriched GO terms (black dashed), or one enriched and one unenriched term (black solid).

**Figure 9:**
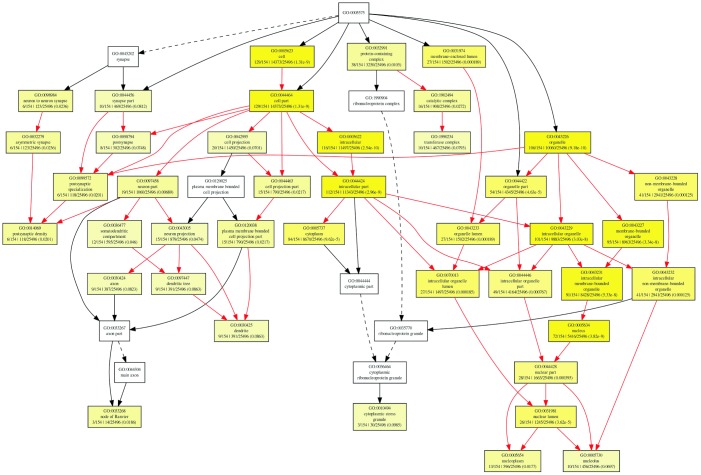
Diagrams of enriched cellular components category of mRNAs targeted of by miRNAs with GOEAST. The yellower the color, the more significant and the smaller p-value it is.

To further investigate the role of circRNAs in isoproterenol induced cardiac hypertrophy, the possible target mRNAs of the 120 miRNAs which were binding to the differentially expressed circRNAs were predicted using DIANA-microT Web server. A total of 7520 target mRNAs were predicted and 156 mRNAs of which were further used for GO analysis by GOEAST software [10]. As shown in Figure 8, among those mRNAs, statistically significantly enriched biological process GO terms were determined, especially those involved in multicellular organism development (GO:0007275), cellular metabolic process (GO:0031323), muscle hypertrophy (GO:0014896), protein modification process (GO:0036211), covalent chromatin modification (GO:0016569), cellular macromolecule localization (GO:0070727), and intracellular protein transport (GO:0006886). Likewise, cellular component category includes significantly enriched target mRNAs in cytoplasm (GO:0005737), intracellular part (GO:0044424), node of Ranvier (GO:0033268), neuron to neuron synapse (GO:0098984), asymmetric synapse (GO:0032279), protein-containing complex (GO:0032991), nuclear lumen (GO:0031981), intracellular membrane-bounded organelle (GO:0043231), and intracellular non-membrane-bounded organelle (GO:0043232) (Figure 9).

**Figure 10:**
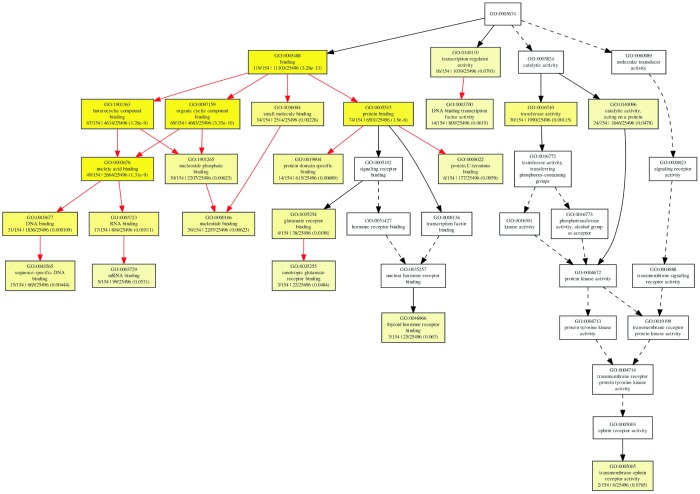
The GOEAST graph of enriched GO terms in the biological process terms for the possible target mRNAs of miRNAs binding to the differentially expressed circRNAs.

But for molecular function ontology, the mRNAs were significantly enriched only in transferase activity (GO:0016740) and binding, which includes DNA binding (GO:0003677), RNA binding (GO:0003723), and protein binding (GO:0005515) (Figure 10), which suggested the differentially expressed circRNAs might play important roles via the binding to DNA, RNA, and protein.

## DISCUSSION

In the present study, we show for the first time that isoproterenol induces profound alterations in circRNA expression in the heart and, most importantly, that the majority of these circRNAs are novel and have not been previously reported before. Specifically, this analysis of the cardiac hypertrophy response induced by isoproterenol suggests that the function of these circRNAs is linked to cardiac hypertrophy.

Although signaling molecules and some epigenetic regulators that exert biological functions in cardiac hypertrophy have been reported in previous studies, the study of circRNAs is just beginning [1, 5, 11]. This study provides strong evidence in support of the role of circRNAs in cardiac hypertrophy. Aside from isoproterenol, transverse aortic constriction also induces changes in the expression profile of circRNAs, indicating that the development of cardiac hypertrophy, rather than stimulus-related factors, could be important for this change. However, interestingly, different cardiac hypertrophy-inducing stimuli result in different circRNA expression profiles. One possible reason for this may be that different stimuli activate different signaling pathways, which induce different circRNA expression profiles and ultimately activate important regulators of cardiac hypertrophy.

Another important issue deserves particular attention: most of the differentially expressed circRNAs identified in this study are novel circRNAs compared with those known circRNAs that have been reported to regulate cardiac development, differentiation, postnatal hypertrophy and heart failure, suggesting that the mechanistic link between circRNAs and cardiac hypertrophy is more complicated than previously thought [7, 9, 12–16]. Indeed, GO and KEGG pathway analysis revealed that the altered expressed circRNAs from isoproterenol treatment were involved into circulatory system, cardiovascular diseases, energy metabolism, and signal transduction pathway. Moreover, functional annotation analyses of predicated target mRNAs of miRNAs which binds to these circRNAs suggested that those mRNAs were related to muscle hypertrophy and cellular metabolic process, which further demonstrated the roles of circRNAs in cardiac hypertrophy. Interestingly, these mRNAs are also involved into protein modification process, covalent chromatin modification, protein-containing complex, indicated that the differentially expressed circRNAs may exert their effects on cardiac hypertrophy through these mechanism.

Beyond these novel circRNAs, we identified 5 known differentially expressed circRNAs, including circRNAs 008365, 015397, 015666, 001283 and 010789, which have been found to be highly expressed in the mammalian brain and be involved in neuronal differentiation [17, 18]. These findings suggest that circRNAs can play multiple roles in physiological and pathological processes.

Indeed, the functions of circRNAs have been found to extend to both development and pathology, especially in the heart [7]. For instance, high-throughput sequencing together with transcriptome analysis have been used to identify the roles of circRNAs in cardiac differentiation [13]. Furthermore, HRCR, a heart-related circRNA, protects the heart from pathological hypertrophy by acting as a molecular sponge for miR-223 [9]. These results identify circRNAs as important regulators in the heart, prompting us to ask whether the novel circRNAs We found in this study could also contribute to cardiac development and postnatal disease. While we have identified several key regulators of cardiac differentiation [19, 20], it remains to be elucidated whether these novel circRNAs exert effects on cardiovascular development and disease through these key regulators.

In summary, to the best of our knowledge, we performed the first comprehensive analysis of circRNA expression by circRNA sequencing in cardiac hypertrophy induced by isoproterenol, and we identified differentially expressed circRNAs involved in cardiac hypertrophy which might facilitate the treatment of cardiac hypertrophy by using circRNA as the potential novel therapeutic targets.

## MATERIALS AND METHODS

### Animal experiments

Eight-week-old mice were infused subcutaneously for 14 days with isoproterenol hydrochloride (ISO, 30 mg/kg/day, Sigma-Aldrich Corporate, St. Louis, MO) or vehicle (saline containing 0.002% ascorbic acid). At the end of the infusion, the mice were anesthetized intraperitoneally with 2.5% Avertin (2 mg/0.01 kg) and sacrificed to collect the ventricles for circRNA analysis. All animal procedures were approved by the Animal Ethics Committee of Zhengzhou University and complied with the Guide for the Care and Use of Laboratory Animals published by the US NIH (2011).

### Echocardiography

The mice were anesthetized with 1.5% isoflurane at the end of the isoproterenol hydrochloride infusion, and echocardiography was performed using a high-resolution Visual Sonics Vevo 2100 (Visual Sonics, Toronto, Ontario, Canada) platform with a 30-MHz mechanical transducer. The fractional shortening (FS), and other parameters, including the interventricular septum thickness at end-diastole (IVSD), the end-diastolic left ventricular posterior wall thickness (LVPWd), end-diastolic left ventricular internal diameter (LVIDd), and end-systolic left ventricular internal diameter (LVIDs), were determined by two-dimensional guided M-mode echocardiography. All results are expressed as the mean ± SEM. Student’s t test was used to compare the two groups. A value of P<0.05 was considered statistically significant.

### RNA-seq analysis

RNA was isolated using the mirVana™ miRNA Isolation Kit (Invitrogen, Grand Island, NY). For next-generation sequencing, 1-3 μg of ribosomal RNA-depleted RNA was purified, fragmented, and then used for first- and second-strand complementary DNA (cDNA) synthesis with random hexamer primers. Next, to construct the RNA-seq libraries, cDNAs were subjected to end repair, the 3' ends were adenylated, the adapters were ligated, and the cDNA templates were enriched. The quality of the prepared libraries was controlled using a Bioanalyzer 2100 system (Agilent, Santa Clara, CA), and RNA-seq was performed using a HiSeq 2500 system (Illumina, San Diego, CA) in paired-end mode.

### Identification and quantification of mouse circRNAs

The raw FASTQ files were filtered for the removal of adapters, reads ≤25, and ribosome RNA reads using Seqtk, and the cleaned sequence data were aligned to the mouse reference genome (Ensembl GRCm38.p4) obtained from UCSC (http://genome.ucsc.edu/) with BWA-MEM. All the unmapped reads were then used to identify circRNAs using CIRI by a backspliced junction with alignment in the reversed orientation (head-to-tail) and breakpoints flanked by GT/AG splice sites. The identified circRNAs were annotated with HOMER and compared to circBase to identify known and novel circRNAs. circRNA expression was normalized by calculating the number of reads spanning the back-spliced junction per million reads of each sample, and the depth of the RNA sequence is 67x.

### Real-time PCR validation

Total RNA was extracted from the ventricles of mice treated with or without isoproterenol, or liver and human skeletal muscle cells, and reverse transcribed into cDNA as described previously [21]. Expression of circRNAs was quantified by SYBR Green Master Mix (Takara, Tokyo, Japan) using the divergent primers while the convergent primers were designed as control. Three biological replicates were performed and a comparative quantification method was used, and the levels of circRNAs were normalized to those of the housekeeping gene GADPH. The sequences of the primers are listed in Table 1.

**Table 1:** Primer sequences.

**circRNA**	**characteristics**	**Forward primer**	**Reverse primer**
circRNA.20948	divergent	TGTCAGATCTTGGTGAAGCAG	TTGGAGTTCCGAGTTTGGAG
	covergent	GCCTTTAAAGTGTGGTGACTCC	AAAGAACTGGTTTGGAACAGC
circRNA.2333	divergent	AACTGTGATCCGGAGAGTGG	GGATGATGATGTCCCCTTTG
	covergent	AGGGGAAAGAACCTGGTGAC	CATAAAGTGCTTTGCCTTGG
circRNA.30270	divergent	GGAGATGGCCTCTTTGTATTTG	CTTCCTCTTGCAGACCATGC
	covergent	TCCTTGGGATCTAGCGACAG	CTACGAGCTGATTGGCTTCC
circRNA.26755	divergent	ACCCTTCTGATTTCTGCTGTAG	CTGTTTCATGCGGTGCAGAG
	covergent	TCAACTCGAAGCTGTCTTGC	ATATCAGCGCAATGGAGGAG
circRNA.20702	divergent	GGGCAATGAGCAGAGTTT	CAAGTCACAGCCAGACATC
	covergent	CGGACACTGATTGAGGCTATC	CTCTCTGCAGAAGCTGTTTCC
circRNA.12000	divergent	AAATGACCCTGAGAGCATGG	GATCTCTGCCCAGGGTTTTC
	covergent	TAAATAACCTGGCCCTGCTG	CAGGTTGTTCTTGGTCTTGG
GAPDH	divergent	CATGGCCTCCAAGGAGTAAG	AGCTACGTGCACCCGTAAAG
	covergent	GTGTTCCTACCCCCAATGTG	ATGTAGGCCATGAGGTCCAC

### Gene ontology and Kyoto Encyclopedia of Genes and Genomes pathway analysis

The parental gene of differentially expressed circRNA between isoproterenol treated and untreated ventricles were analyzed their potential biological roles through gene ontology (GO) analysis and Kyoto Encyclopedia of Genes and Genomes (KEGG) pathway analysis. The ontology includes biological process, cellular component, and molecular function ontologies, while the KEGG pathway consists of six top categories covering organismal systems, human diseases, metabolism, genetic information processing, cellular processes, and environmental information processing.

### Prediction of miRNA binding to circRNAs, mRNA targets of miRNA, construction of circRNA-miRNA interaction network, and Gene Ontology analysis

miRNA targets of differentially expressed circRNAs between isoproterenol treated and untreated ventricles were predicted by miRanda (http://www.microrna.org/microrna/home.do). Meanwhile, the circRNA-miRNA regulatory network was constructed and displayed by Cytoscape V3.7.0 (Institute of Systems Biology, WA, USA). Next, the possible target mRNAs of the obtained miRNAs were further predicted using DIANA-microT Web server, xand the annotation of functions of predicted target mRNAs were performed with the GO Enrichment Analysis Software Toolkit (GOEAST) [10].

## Supplementary Material

Supplementary Table 1
