# Supplementary material for: Circular RNA expression in isoproterenol hydrochloride-induced cardiac hypertrophy
Source: Aging (Albany NY). 2020 Feb 5;12(3):2530–44. doi: 10.18632/aging.102761 (PMC7041747; doi:10.18632/aging.102761)
Supplement: Supplementary Table 1 [file aging-12-102761-s001..pdf]

SUPPLEMENTAL MATERIAL

Table 1: Altered expression of circRNAs between ventricles without or with isoproterenol treatment

circRNA_id	g2	g1	log2FC	P value	updown
circRNA.2333	0.209357712	8.711299301	-5.3788459	0.002228719	down
circRNA.26528	0.058826481	1.372269239	-4.543954	0.034092358	-
circRNA.24264	0.583080433	7.253504394	-3.6369114	0.006608716	-
circRNA.38703	0.075265616	0.841281541	-3.4825258	0.00185158	-
circRNA.20095	0.083724409	0.853705056	-3.3500175	0.000872032	-
circRNA.34475	0.050177077	0.499779021	-3.31619	0.019712935	-
circRNA.18771	4.504629504	44.02125513	-3.2887199	0.027482057	-
circRNA.33115	0.075265616	0.582255541	-2.9515896	0.007083741	-
circRNA.17043	0.075265616	0.532727399	-2.8233346	0.009293492	-
circRNA.14835	0.058826481	0.412921246	-2.811329	0.041830136	-
circRNA.16728	0.139540682	0.898957518	-2.6875672	0.010103527	-
circRNA.27779	0.083724409	0.490566501	-2.5507285	0.034880625	-
circRNA.18550	0.362805773	1.98339294	-2.4507012	0.013291333	-
circRNA.29262	0.100354155	0.544370323	-2.4394881	0.028796457	-
circRNA.10747	2.148926502	10.87637304	-2.3395095	0.043372295	-
circRNA.30772	0.111632546	0.529741409	-2.2465306	0.027229056	-
circRNA.16026	0.10599335	0.501624296	-2.2426335	0.019089022	-
circRNA.38766	0.250885386	1.106504526	-2.140909	0.024328111	-
circRNA.10841	0.470685447	1.859456999	-1.9820462	0.014563451	-
circRNA.6475	0.150531232	0.590657926	-1.97226	0.048230955	-
circRNA.26052	0.114642753	0.438607955	-1.9357868	0.040812577	-
circRNA.2577	0.167448818	0.638489768	-1.9309433	0.050719799	-
circRNA.21430	0.163505337	0.622495134	-1.9287248	0.038704453	-
circRNA.1850	0.192918577	0.666352331	-1.7882931	0.034969367	-
circRNA.31323	0.247420356	0.831004337	-1.7478918	0.019594662	-
circRNA.38517	0.242714434	0.728002953	-1.5846844	0.039537461	-
circRNA.25682	0.409229979	1.136241909	-1.4732863	0.036205364	-
circRNA.29985	5.548383713	14.5346441	-1.3893563	0.043353402	-
circRNA.2554	0.350403498	0.77801065	-1.1507727	0.050865163	-
circRNA.12000	10.40622861	3.309371493	1.65281815	0.013207787	up
circRNA.38217	4.052829274	1.28829188	1.65346991	0.020568356	-
circRNA.35512	3.506447018	1.073556148	1.70761228	0.034559613	-
circRNA.30270	49.06250486	13.82715216	1.82711684	0.008346543	-
circRNA.37718	1.702700086	0.478367992	1.83163157	0.03554703	-
circRNA.29624	2.876078855	0.797169794	1.85114428	0.028701768	-
circRNA.21764	3.538010794	0.96156725	1.87947879	0.022105918	-

circRNA.6709	1.848428165	0.472141463	1.96900789	0.027306632	-
circRNA.34138	1.076968095	0.264187845	2.02733952	0.024055385	-
circRNA.6764	2.590980927	0.619178565	2.06507096	0.049353724	-
circRNA.22584	0.912793697	0.214180147	2.09146407	0.046760987	-
circRNA.34972	1.705998135	0.394826787	2.11132429	0.023026863	-
circRNA.36934	0.650629919	0.150353671	2.11347707	0.050317389	-
circRNA.11111	5.998267424	1.37570524	2.12437445	0.010947328	-
circRNA.28597	0.897859666	0.204967628	2.1310939	0.034683699	-
circRNA.6391	1.1811685	0.268794104	2.13564139	0.02107984	-
circRNA.22198	0.942397547	0.214180147	2.13751102	0.036870665	-
circRNA.34551	0.904334904	0.204967628	2.14146107	0.046107448	-
circRNA.13392	0.677223562	0.150353671	2.17127207	0.049732102	-
circRNA.14992	0.784912626	0.17305439	2.18130653	0.047902566	-
circRNA.31880	1.164348145	0.255305905	2.18922368	0.04905326	-
circRNA.26489	0.796191017	0.17305439	2.20188906	0.037855265	-
circRNA.13822	0.82240344	0.17766065	2.21072213	0.034061639	-
circRNA.37828	0.828614465	0.17766065	2.22157683	0.03504049	-
circRNA.21173	2.115013875	0.451061014	2.22927263	0.009725382	-
circRNA.22411	0.900679263	0.191479429	2.23382404	0.046905041	-
circRNA.35990	2.106123889	0.446785334	2.23693657	0.050516769	-
circRNA.10808	1.032597194	0.209573888	2.30074671	0.028618755	-
circRNA.37818	2.131476639	0.425119755	2.32591204	0.017156187	-
circRNA.18628	2.598027995	0.51463294	2.33580127	0.019254377	-
circRNA.16555	0.614741441	0.118440433	2.37581807	0.050566006	-
circRNA.37054	0.63268568	0.118440433	2.41732727	0.03685708	-
circRNA.28835	1.365056454	0.255305905	2.41866181	0.029679815	-
circRNA.17527	0.747328434	0.127652952	2.54951551	0.028085502	-
circRNA.38102	0.864312334	0.145747412	2.56808249	0.014166502	-
circRNA.25865	0.650629919	0.109227914	2.57449558	0.047318244	-
circRNA.12939	0.659279323	0.109227914	2.59354824	0.046586091	-
circRNA.1234	0.924072088	0.150353671	2.61964531	0.013144468	-
circRNA.23614	0.743003732	0.118440433	2.64920779	0.014202871	-
circRNA.32393	0.769118923	0.118440433	2.69904502	0.02479629	-
circRNA.17215	0.722049285	0.109227914	2.72475572	0.044384693	-
circRNA.30960	0.64348562	0.095739714	2.7487185	0.031216828	-
circRNA.15924	4.529055548	0.670178	2.756594	0.034058693	-
circRNA.31987	0.879055755	0.127652952	2.78372777	0.0211165	-
circRNA.16584	0.879627585	0.127652952	2.78466594	0.030244308	-
circRNA.21198	0.82259405	0.118440433	2.79601897	0.007727062	-
circRNA.37485	0.668574159	0.095739714	2.80389819	0.025585865	-
circRNA.38237	0.768282881	0.109227914	2.81429602	0.023863014	-
circRNA.5547	0.588338409	0.081920935	2.84434213	0.050636957	-
circRNA.26357	0.784912626	0.109227914	2.84519048	0.025531089	-

circRNA.17037	0.596128141	0.081920935	2.86331839	0.037994821	-
circRNA.26656	0.611252782	0.081920935	2.89946503	0.046776048	-
circRNA.32916	0.614932051	0.081920935	2.90812291	0.03828305	-
circRNA.4619	0.487315193	0.063826476	2.9326283	0.037754881	-
circRNA.11257	3.627804454	0.474317318	2.93517225	0.036032586	-
circRNA.4109	0.638515486	0.081920935	2.96241752	0.031454118	-
circRNA.25385	4.451235684	0.567881177	2.97054489	3.43E-05	-
circRNA.24182	0.751174684	0.095739714	2.97195904	0.014263373	-
circRNA.30834	0.512403731	0.063826476	3.00505408	0.037280327	-
circRNA.15820	0.518042927	0.063826476	3.02084474	0.027590235	-
circRNA.22731	0.668764769	0.081920935	3.02919475	0.031017572	-
circRNA.22668	0.674022744	0.081920935	3.04049318	0.033332914	-
circRNA.5474	4.560214475	0.542194468	3.07221938	0.016701883	-
circRNA.19624	0.540121258	0.063826476	3.08105642	0.034455699	-
circRNA.4269	0.540311868	0.063826476	3.08156546	0.026946544	-
circRNA.28719	0.696482295	0.081920935	3.08778259	0.019013909	-
circRNA.38016	7.385924504	0.847508071	3.1234795	0.017552305	-
circRNA.17230	1.117659727	0.127652952	3.13018221	0.006458267	-
circRNA.30082	0.85200729	0.095739714	3.15367637	0.00464736	-
circRNA.14333	0.495964596	0.054613957	3.18289555	0.038880327	-
circRNA.2923	0.579689005	0.063826476	3.18305222	0.016110173	-
circRNA.12806	0.89955538	0.095739714	3.2320227	0.004750289	-
circRNA.20702	3.412715305	0.335275991	3.34749899	0.00144822	-
circRNA.1525	0.556105571	0.054613957	3.3480172	0.023842303	-
circRNA.31704	0.593308543	0.054613957	3.44144096	0.01527625	-
circRNA.33287	0.699683113	0.063826476	3.45447477	0.012350204	-
circRNA.33036	1.058642636	0.095739714	3.46695435	0.004590283	-
circRNA.3052	0.92125249	0.081920935	3.49129252	0.004106933	-
circRNA.5016	0.943066609	0.081920935	3.52505558	0.003221031	-
circRNA.33776	0.646305218	0.054613957	3.56487405	0.011365271	-
circRNA.3968	0.675909068	0.054613957	3.62948758	0.009562198	-
circRNA.34143	1.237677464	0.095739714	3.69237409	0.017906775	-
circRNA.37573	0.717724583	0.054613957	3.71608874	0.007575297	-
circRNA.27850	1.806375919	0.136534892	3.72575657	0.021444763	-
circRNA.22130	0.850883406	0.063826476	3.73673455	0.002227223	-
circRNA.19255	0.891575038	0.054613957	4.02901463	0.001660401	-
circRNA.33783	1.049705391	0.062206206	4.07678214	0.011923821	-
circRNA.10555	3.728540967	0.155515516	4.58348076	0.002211948	-
circRNA.16350	2.730056368	0.095739714	4.83366943	0.002219934	-
circRNA.34921	14.89615986	0	Inf	4.54E-06	up
circRNA.16181	6.888157244	0	Inf	2.46E-05	-
circRNA.14424	4.309105569	0	Inf	3.44E-05	-
circRNA.18244	4.143854545	0	Inf	4.18E-05	-

circRNA.14819	11.93953903	0	Inf	7.60E-05	-
circRNA.24516	2.868390206	0	Inf	9.43E-05	-
circRNA.21253	0.763767569	0	Inf	0.000140265	-
circRNA.18956	0.7858459	0	Inf	0.00016698	-
circRNA.1576	2.045979062	0	Inf	0.000187063	-
circRNA.30349	0.735859433	0	Inf	0.000191176	-
circRNA.17764	228.7173569	0	Inf	0.000212898	-
circRNA.35201	1.826252603	0	Inf	0.000286882	-
circRNA.34085	0.912315245	0	Inf	0.000328846	-
circRNA.682	0.887226707	0	Inf	0.000383705	-
circRNA.968	0.67985255	0	Inf	0.000425723	-
circRNA.11007	0.788020065	0	Inf	0.000572611	-
circRNA.33967	0.654764011	0	Inf	0.00061115	-
circRNA.2261	14.50072749	0	Inf	0.000702513	-
circRNA.33657	16.05666472	0	Inf	0.000708784	-
circRNA.23859	0.724868883	0	Inf	0.000761668	-
circRNA.24352	0.664727908	0	Inf	0.000957498	-
circRNA.10966	8.853385343	0	Inf	0.000977784	-
circRNA.17212	0.685203904	0	Inf	0.001043582	-
circRNA.554	8.22904067	0	Inf	0.001114473	-
circRNA.28477	7.852712591	0	Inf	0.001161713	-
circRNA.20959	0.578183901	0	Inf	0.001167598	-
circRNA.254	6.88269824	0	Inf	0.001181061	-
circRNA.36389	6.623374198	0	Inf	0.001338563	-
circRNA.36181	0.744054014	0	Inf	0.001474796	-
circRNA.12132	5.581627278	0	Inf	0.001501087	-
circRNA.12197	0.565400406	0	Inf	0.001507818	-
circRNA.12989	0.550275765	0	Inf	0.00154402	-
circRNA.5168	0.584204317	0	Inf	0.001617606	-
circRNA.24219	0.615386873	0	Inf	0.00162014	-
circRNA.2467	1.419625268	0	Inf	0.00162611	-
circRNA.8073	0.917142028	0	Inf	0.001733478	-
circRNA.22016	0.607406532	0	Inf	0.001765758	-
circRNA.14488	0.912626716	0	Inf	0.001777449	-
circRNA.670	4.691556724	0	Inf	0.001845244	-
circRNA.488	0.568410614	0	Inf	0.00186524	-
circRNA.31837	4.297853004	0	Inf	0.001934214	-
circRNA.853	0.538806764	0	Inf	0.002243357	-
circRNA.27980	0.521053135	0	Inf	0.002257125	-
circRNA.2354	3.612749563	0	Inf	0.002406809	-
circRNA.28647	0.516537823	0	Inf	0.002457593	-
circRNA.17308	0.850404955	0	Inf	0.002473923	-
circRNA.29611	0.525377836	0	Inf	0.002581036	-

circRNA.29000	3.361864176	0	Inf	0.002597015	-
circRNA.9512	0.581839542	0	Inf	0.002603231	-
circRNA.34334	2.734997366	0	Inf	0.003147043	-
circRNA.31009	2.651272957	0	Inf	0.003235718	-
circRNA.11189	0.563704692	0	Inf	0.003330352	-
circRNA.3951	2.567548548	0	Inf	0.003398411	-
circRNA.23506	0.482990491	0	Inf	0.003595846	-
circRNA.34058	2.458676786	0	Inf	0.003723002	-
circRNA.32951	0.562771419	0	Inf	0.00382109	-
circRNA.33252	0.465046252	0	Inf	0.004272476	-
circRNA.2292	0.463541148	0	Inf	0.004751439	-
circRNA.13505	2.032171629	0	Inf	0.004779684	-
circRNA.31197	0.494650102	0	Inf	0.004850845	-
circRNA.12	0.755763598	0	Inf	0.004898493	-
circRNA.12854	1.73538118	0	Inf	0.005229616	-
circRNA.4005	0.472190551	0	Inf	0.005351821	-
circRNA.14353	1.856551859	0	Inf	0.005427381	-
circRNA.28891	1.731109165	0	Inf	0.005865933	-
circRNA.6508	0.437138115	0	Inf	0.006397131	-
circRNA.35693	1.590763774	0	Inf	0.006514797	-
circRNA.35991	0.432813414	0	Inf	0.006710342	-
circRNA.25647	0.432813414	0	Inf	0.006711277	-
circRNA.2690	0.434318518	0	Inf	0.006951306	-
circRNA.7102	0.447292623	0	Inf	0.007384819	-
circRNA.3543	0.444473025	0	Inf	0.007829409	-
circRNA.7167	0.414869175	0	Inf	0.008095095	-
circRNA.6280	1.379869625	0	Inf	0.008212505	-
circRNA.37021	0.474341088	0	Inf	0.008329507	-
circRNA.1974	0.413364071	0	Inf	0.008690796	-
circRNA.29547	0.626019832	0	Inf	0.008811002	-
circRNA.23302	0.494078272	0	Inf	0.009014214	-
circRNA.2666	0.425669114	0	Inf	0.009267364	-
circRNA.2280	1.304604009	0	Inf	0.009308784	-
circRNA.4177	0.647238491	0	Inf	0.009580024	-
circRNA.16828	1.117703133	0	Inf	0.009825187	-
circRNA.23573	0.410544473	0	Inf	0.009843585	-
circRNA.7136	0.410544473	0	Inf	0.00984578	-
circRNA.2537	0.642077747	0	Inf	0.010290133	-
circRNA.20490	0.382636336	0	Inf	0.010424233	-
circRNA.1488	0.439767103	0	Inf	0.01043463	-
circRNA.22096	1.179161316	0	Inf	0.010596871	-
circRNA.37409	0.406219771	0	Inf	0.010637912	-
circRNA.25823	0.38414144	0	Inf	0.010747206	-

circRNA.30934	0.379816739	0	Inf	0.010787117	-
circRNA.20327	0.447938055	0	Inf	0.011400139	-
circRNA.6614	1.128984238	0	Inf	0.011788492	-
circRNA.16295	0.381131233	0	Inf	0.011810201	-
circRNA.37026	0.400580576	0	Inf	0.011848942	-
circRNA.26556	0.422204084	0	Inf	0.011856786	-
circRNA.10451	1.000050172	0	Inf	0.011949158	-
circRNA.11331	0.381321843	0	Inf	0.012054739	-
circRNA.2453	0.381321843	0	Inf	0.012058696	-
circRNA.27971	0.42435462	0	Inf	0.012888659	-
circRNA.2435	0.389780636	0	Inf	0.013033752	-
circRNA.35528	1.00469291	0	Inf	0.013205422	-
circRNA.27111	0.38395083	0	Inf	0.013373604	-
circRNA.16681	0.397760978	0	Inf	0.014313337	-
circRNA.34397	0.911810451	0	Inf	0.014342801	-
circRNA.28658	0.911810451	0	Inf	0.014347392	-
circRNA.18924	0.378311635	0	Inf	0.015096371	-
circRNA.31586	0.3547282	0	Inf	0.015330624	-
circRNA.2021	0.3547282	0	Inf	0.015333968	-
circRNA.26186	0.357547798	0	Inf	0.015909872	-
circRNA.11563	0.357547798	0	Inf	0.015910066	-
circRNA.15628	0.357547798	0	Inf	0.015910206	-
circRNA.2091	0.920968501	0	Inf	0.016389562	-
circRNA.17187	0.350403498	0	Inf	0.016529602	-
circRNA.18538	0.376997141	0	Inf	0.016530337	-
circRNA.25245	0.387151648	0	Inf	0.016858572	-
circRNA.11530	0.351908602	0	Inf	0.017035537	-
circRNA.35221	0.518902599	0	Inf	0.017089903	-
circRNA.11307	0.356233304	0	Inf	0.017186868	-
circRNA.19528	0.382445726	0	Inf	0.017490037	-
circRNA.17952	0.382445726	0	Inf	0.017496908	-
circRNA.18672	0.356042694	0	Inf	0.017609115	-
circRNA.16431	0.356042694	0	Inf	0.017615708	-
circRNA.36428	0.360367396	0	Inf	0.018645881	-
circRNA.13290	0.376806531	0	Inf	0.01883334	-
circRNA.24626	0.372672439	0	Inf	0.01909115	-
circRNA.35137	0.373986933	0	Inf	0.019095498	-
circRNA.1928	0.878098852	0	Inf	0.019244289	-
circRNA.38030	0.379626129	0	Inf	0.019803506	-
circRNA.35184	0.589081072	0	Inf	0.019988619	-
circRNA.24785	0.487984254	0	Inf	0.020425204	-
circRNA.5731	0.358862292	0	Inf	0.021043208	-
circRNA.32754	0.329639661	0	Inf	0.022739851	-

circRNA.20133	0.329639661	0	Inf	0.022750227	-
circRNA.34931	0.32531496	0	Inf	0.023185319	-
circRNA.3868	0.32531496	0	Inf	0.02318918	-
circRNA.36516	0.519069579	0	Inf	0.023223251	-
circRNA.29346	0.802833236	0	Inf	0.023446599	-
circRNA.36924	0.802833236	0	Inf	0.023447126	-
circRNA.37509	0.326820064	0	Inf	0.023745674	-
circRNA.15721	0.328134558	0	Inf	0.023824661	-
circRNA.18640	0.465427472	0	Inf	0.024963969	-
circRNA.12011	0.465427472	0	Inf	0.024982848	-
circRNA.16613	0.45706591	0	Inf	0.025416935	-
circRNA.23624	0.460076118	0	Inf	0.025516105	-
circRNA.10455	0.482609271	0	Inf	0.025578661	-
circRNA.31331	0.705917768	0	Inf	0.025596684	-
circRNA.37321	0.357357188	0	Inf	0.026158205	-
circRNA.22940	0.490780223	0	Inf	0.026763772	-
circRNA.10390	0.752656159	0	Inf	0.026797789	-
circRNA.29556	0.323809856	0	Inf	0.026963597	-
circRNA.35346	0.330954155	0	Inf	0.027346025	-
circRNA.25381	0.330954155	0	Inf	0.027349373	-
circRNA.22599	0.328325168	0	Inf	0.02880533	-
circRNA.37916	0.324000466	0	Inf	0.028837035	-
circRNA.23337	0.326629454	0	Inf	0.028989933	-
circRNA.36627	0.335469467	0	Inf	0.029455508	-
circRNA.15322	0.72756762	0	Inf	0.029817451	-
circRNA.13143	0.31817066	0	Inf	0.030466566	-
circRNA.33319	0.31817066	0	Inf	0.030493077	-
circRNA.13367	0.31817066	0	Inf	0.03049383	-
circRNA.13996	0.336783961	0	Inf	0.030829437	-
circRNA.28225	0.319675764	0	Inf	0.031230708	-
circRNA.28284	0.319675764	0	Inf	0.031231289	-
circRNA.20997	0.335278857	0	Inf	0.031508638	-
circRNA.3558	0.647091288	0	Inf	0.031932169	-
circRNA.25579	0.435178189	0	Inf	0.032259019	-
circRNA.22684	0.443422743	0	Inf	0.032269916	-
circRNA.37206	0.360176785	0	Inf	0.032728536	-
circRNA.20995	0.426792998	0	Inf	0.033604418	-
circRNA.24543	0.300226421	0	Inf	0.033759321	-
circRNA.35582	0.300226421	0	Inf	0.033761454	-
circRNA.16403	0.300226421	0	Inf	0.033768383	-
circRNA.12683	0.300226421	0	Inf	0.033776933	-
circRNA.24379	0.677390543	0	Inf	0.034028789	-
circRNA.13105	0.677390543	0	Inf	0.034038509	-

circRNA.27861	0.301731525	0	Inf	0.034502104	-
circRNA.22052	0.301731525	0	Inf	0.034517541	-
circRNA.19909	0.301731525	0	Inf	0.034537021	-
circRNA.30877	0.297406823	0	Inf	0.035103514	-
circRNA.26241	0.297406823	0	Inf	0.03510465	-
circRNA.4847	0.415250395	0	Inf	0.035462526	-
circRNA.38638	0.652302004	0	Inf	0.037752777	-
circRNA.24504	0.295901719	0	Inf	0.03779884	-
circRNA.35502	0.306056227	0	Inf	0.038142898	-
circRNA.26837	0.641887137	0	Inf	0.038295556	-
circRNA.32159	0.439479262	0	Inf	0.038848128	-
circRNA.15104	0.298911927	0	Inf	0.039545797	-
circRNA.38768	0.399744533	0	Inf	0.04049439	-
circRNA.35435	0.399744533	0	Inf	0.040503805	-
circRNA.38721	0.303236629	0	Inf	0.040799085	-
circRNA.27181	0.332268649	0	Inf	0.040815822	-
circRNA.1362	0.396065264	0	Inf	0.041097308	-
circRNA.10412	0.307370721	0	Inf	0.041484317	-
circRNA.16950	0.307370721	0	Inf	0.041506264	-
circRNA.7164	0.294587226	0	Inf	0.041569538	-
circRNA.22516	0.393245666	0	Inf	0.041853741	-
circRNA.17516	0.401704459	0	Inf	0.042104861	-
circRNA.11922	0.308875825	0	Inf	0.04222476	-
circRNA.28306	0.301540915	0	Inf	0.043432549	-
circRNA.34714	0.613979001	0	Inf	0.043574106	-
circRNA.31253	0.305865617	0	Inf	0.044331434	-
circRNA.28659	0.305865617	0	Inf	0.044341781	-
circRNA.38777	0.390161856	0	Inf	0.044985677	-
circRNA.36724	0.307561331	0	Inf	0.045819395	-
circRNA.23318	0.27814809	0	Inf	0.047001698	-
circRNA.15468	0.280967688	0	Inf	0.047639753	-
circRNA.274	0.290262524	0	Inf	0.048818788	-
circRNA.12779	0.408775157	0	Inf	0.049406801	-
circRNA.20440	0.577036388	0	Inf	0.049610796	-
circRNA.7101	0.272318285	0	Inf	0.050491798	-
circRNA.28960	0.272318285	0	Inf	0.050492367	-
circRNA.16353	0.272318285	0	Inf	0.050503772	-
circRNA.24321	0.272318285	0	Inf	0.050511806	-
circRNA.36712	0	2.2379912	#NAME?	1.84E-09	down
circRNA.26755	0	1.223400719	#NAME?	1.52E-06	-
circRNA.20948	0	7.472384347	#NAME?	7.61E-06	-
circRNA.4633	0	0.746907547	#NAME?	2.66E-05	-
circRNA.23053	0	3.125702884	#NAME?	6.92E-05	-

circRNA.7831	0	0.85757721	#NAME?	9.00E-05	-
circRNA.2071	0	1.944919509	#NAME?	9.95E-05	-
circRNA.12926	0	23.86629916	#NAME?	0.000237528	-
circRNA.16992	0	24.12640802	#NAME?	0.000253731	-
circRNA.30490	0	1.172731863	#NAME?	0.00029488	-
circRNA.38418	0	12.69774498	#NAME?	0.000356485	-
circRNA.14727	0	11.05932627	#NAME?	0.000394016	-
circRNA.27215	0	5.520990194	#NAME?	0.000802369	-
circRNA.306	0	4.883187203	#NAME?	0.000822953	-
circRNA.21410	0	0.951799145	#NAME?	0.000853929	-
circRNA.36425	0	0.52675542	#NAME?	0.00093022	-
circRNA.5629	0	3.576856868	#NAME?	0.001019825	-
circRNA.2635	0	3.413372306	#NAME?	0.001201437	-
circRNA.31076	0	3.510456193	#NAME?	0.001345029	-
circRNA.18068	0	0.760065167	#NAME?	0.001425881	-
circRNA.26973	0	2.512242017	#NAME?	0.001759349	-
circRNA.35412	0	2.266479211	#NAME?	0.001971844	-
circRNA.9764	0	0.954966739	#NAME?	0.002036908	-
circRNA.35563	0	1.337433438	#NAME?	0.002625125	-
circRNA.36664	0	1.306330335	#NAME?	0.002724573	-
circRNA.29249	0	1.181917922	#NAME?	0.00304778	-
circRNA.23774	0	1.819054573	#NAME?	0.003305916	-
circRNA.11857	0	1.057505509	#NAME?	0.003461299	-
circRNA.25653	0	1.755228096	#NAME?	0.003530815	-
circRNA.30883	0	0.545405464	#NAME?	0.003755145	-
circRNA.34291	0	0.995299302	#NAME?	0.00379198	-
circRNA.14299	0	0.43352214	#NAME?	0.00386671	-
circRNA.4413	0	0.933093096	#NAME?	0.004214386	-
circRNA.33202	0	0.380197873	#NAME?	0.005016305	-
circRNA.29693	0	1.372269239	#NAME?	0.005390539	-
circRNA.33586	0	1.119586116	#NAME?	0.006195147	-
circRNA.11871	0	0.715371374	#NAME?	0.006276556	-
circRNA.4030	0	0.622062064	#NAME?	0.007981571	-
circRNA.25645	0	0.569170867	#NAME?	0.008922893	-
circRNA.5774	0	0.34909477	#NAME?	0.009119519	-
circRNA.1233	0	0.559855858	#NAME?	0.009331258	-
circRNA.30463	0	0.989310382	#NAME?	0.010207092	-
circRNA.33137	0	0.528752754	#NAME?	0.010531864	-
circRNA.6805	0	0.542194468	#NAME?	0.011235195	-
circRNA.31865	0	0.925483905	#NAME?	0.01237453	-
circRNA.14970	0	0.925483905	#NAME?	0.012378285	-
circRNA.25807	0	0.478367992	#NAME?	0.014618154	-
circRNA.28421	0	0.420993051	#NAME?	0.016201055	-

circRNA.24475	0	0.70998144	#NAME?	0.017125867	-
circRNA.28536	0	0.326949636	#NAME?	0.020481317	-
circRNA.6904	0	0.655367483	#NAME?	0.021772135	-
circRNA.6049	0	0.460273533	#NAME?	0.022579478	-
circRNA.28808	0	0.2663637	#NAME?	0.024739362	-
circRNA.32941	0	0.275576219	#NAME?	0.024928161	-
circRNA.40	0	0.271780094	#NAME?	0.025016398	-
circRNA.29316	0	0.378022018	#NAME?	0.032363453	-
circRNA.16905	0	0.546139569	#NAME?	0.032909208	-
circRNA.463	0	0.546139569	#NAME?	0.03291753	-
circRNA.28614	0	0.288254283	#NAME?	0.035477625	-
circRNA.16470	0	0.288254283	#NAME?	0.035492881	-
circRNA.15537	0	0.268539555	#NAME?	0.038039402	-
circRNA.22154	0	0.355321299	#NAME?	0.040340997	-
circRNA.555	0	0.244473116	#NAME?	0.043095318	-
circRNA.28755	0	0.244473116	#NAME?	0.043101123	-
circRNA.27101	0	0.34610878	#NAME?	0.043388257	-
circRNA.17265	0	0.34610878	#NAME?	0.043406613	-
circRNA.26779	0	0.364533819	#NAME?	0.043502039	-
circRNA.11793	0	0.283978603	#NAME?	0.044903124	-

g1: control; g2: isoproterenol treatment; FC: fold-change.
